# Innovation in Learning: Critical Review of the Literature Is Absolutely Necessary for the Modern Medical Student

**DOI:** 10.7759/cureus.34055

**Published:** 2023-01-22

**Authors:** Carl Hoegerl, Adam Barton, Raena Pettitt

**Affiliations:** 1 Neurology, Centra Health System, Lynchburg, USA; 2 Internal Medicine and Neurology, Liberty University College of Osteopathic Medicine, Lynchburg, USA; 3 Internal Medicine, Liberty University College of Osteopathic Medicine, Lynchburg, USA; 4 Family Medicine, Liberty University College of Osteopathic Medicine, Lynchburg, USA

**Keywords:** osteopathic medicine, literature review, critical review, medical education, journal club

## Abstract

The concept of a journal club has been around for decades and is a useful tool to help teach residents about evidence-based medicine. Although some students may be exposed to the concept during their third or fourth year of medical school, many do not have exposure to this until their residency. This innovation report describes a program to help introduce first and second-year medical students to a team-based approach to journal clubs so that they can apply principles of evidence-based practice early on in their medical school training.

In the Fall of 2020, a faculty and student effort led by members of the internal medicine and family medicine interest groups met to form a team-based program approach to the journal club focusing on first and second-year students. Teams of students were formed, and they were allowed to pick an article to review. They presented their findings to a group of students and faculty members via online meetings (due to coronavirus disease 2019 (COVID-19) restrictions).

This program has been introduced and done for two academic years. During that time, over 50 students participated in the program along with six faculty members. Countless students and faculty have been an audience to the presentations that have been done. The program is entering its third year of providing an engaging avenue for students to gain experience in analyzing articles and making presentations. The journal club program has and continues to be praised by faculty and students for its unique approach and style to a decade-old tradition.

We recommend that medical schools and other health-science programs across the country consider introducing a program like this to help stimulate critical discussions in an era of increasingly published medical literature. It is critical that students be given a chance to apply evidence-based practices early on in their medical education careers.

## Editorial

Background and importance

The use of journal clubs is thought to have started with Sir William Osler at McGill University in 1875 [1]. Ever since then, journal clubs have been used to help educate residents about how to critically appraise research literature. This ability for a student to be able to critically appraise the literature is not only important for accrediting bodies but also for future careers. The use and benefits of journal clubs in medical schools for first and second-year students have not been well-documented. The majority of medical schools within the United States focus on lecture-based, case-based, or problem-based learning for pre-clinical years. Often, schools do not do a very good job of training students to critically appraise a paper, which makes them ill-prepared for residency [2]. Although the coronavirus disease 2019 (COVID-19) pandemic has forced many institutions to do some new and innovative methods for journal clubs, such as the use of Twitter, there still is a dearth of literature showing how journal clubs can benefit students in the preclinical years [3,4]. However, many students do not often get the experience of analyzing and presenting medical literature in a manner that prepares them for a journal club in residency. Residency programs use journal clubs as a way for residents to learn the proper way to interpret, examine, present, and discuss scientific papers among a healthcare team [5]. Incorporating journal clubs earlier within medical education helps to build a foundation in areas such as communication, analytical skills, and confidence in presenting scientific information. These skills will undoubtedly be used throughout a career as a physician. This paper aims to discuss our experience and methods of conducting a journal club at the Liberty University College of Osteopathic Medicine (LUCOM) for first- and second-year medical students. There is great importance in trying to instill the skills necessary to appraise scientific literature that is constantly changing. The concept of a journal club is a great way to apply knowledge and gain the skills necessary to be life-long learners and critics. The introduction of this concept earlier in the career of a physician, the better.

Our approach

Journal clubs at LUCOM are currently hosted by two student organizations, the Family Medicine and Internal Medicine interest groups. In the first year of the program in 2020, there was one session held each semester. During each session, there were four teams of six students. Each team analyzed one article. The teams were introduced to the concept of journal clubs and given instructions on how to analyze each article based on specific criteria. Students were instructed to break down each article into relevant sections, including background, methods, results, and discussion. It was expected that they critique each part of the article including the validity of findings, bias, study design, and statistical analysis. Students were expected to defend their findings and arguments. This project was initially started to be done virtually during the COVID-19 lockdown but has been continued in person since.

Results

Since the creation of both journal clubs, over 50 students and six faculty, five of who were clinicians and one biomedical professor, have participated and added to discussions. Each year participation and attendance have increased. Specifically, during the 2021-2022 academic year, the Family Medicine interest group held two article review sessions that were led by students, the January session included eight student presenters spread across three groups and the March session included four student presenters spread across two groups. During the same year, the Internal Medicine interest group hosted two meetings with a total of three articles being presented from a total of five students. The journal article topics were chosen to mirror the current subject matter that the students were currently learning in the curriculum. This has turned out to be a great way to augment the primary curriculum of medical students with additional “outside-the-classroom” learning opportunities. The family medicine interest group created a sign-up sheet for other clubs to participate in and there are currently several other clubs participating in the project. The number of students participating continues to grow.

What makes this project particularly unique is the grassroots efforts by medical students to incorporate this important concept of journal clubs in a co-curricular manner using student interest groups as the basis for implementation. It gets students engaged in the journal clubs and motivates them to learn it well. The experience hopefully will get them excited about evidence-based medicine for a lifetime.

In the future, we plan on continuing to host journal clubs for first- and second-year medical students at LUCOM within the Family Medicine and Internal Medicine interest groups. However, we also plan to conduct a Likert scale questionnaire for students who participate in the journal club meetings. This will allow students to give feedback on how our journal clubs operate and will also assess whether the journal clubs meet our objectives. We also plan to start a journal club outside of specialty interest groups, for the purpose of learning, practicing, and developing skills useful for a journal club that will be experienced in residency. We believe it would also be beneficial to add a journal club activity within the Population-Based Medicine (PBM) course, which is taken by first-year students. This activity would complement this course because it directly addresses statistics, scientific design, population health, and interpreting scientific literature. Therefore, incorporating an activity where groups of students critically analyze medical research papers and present their findings to their peers and professors could be of terrific value. Approaching an activity like this from both an extracurricular and curricular standpoint, we feel, would be very beneficial to our students. It is a great way to instill skills in these students that will last a lifetime.
